# Impact of Soil-Based Insulation on Ultrahigh-Resolution Fiber-Optic Interferometry

**DOI:** 10.3390/s23010259

**Published:** 2022-12-27

**Authors:** Nabil Md Rakinul Hoque, Lingze Duan

**Affiliations:** Department of Physics and Astronomy, University of Alabama in Huntsville, Huntsville, AL 35899, USA

**Keywords:** optical interferometry, fiber optics, Mach-Zehnder interferometer, Fabry-Perot cavity, acoustic insulation, thermal insulation, soil

## Abstract

High resolution optical interferometry often requires thermal and acoustic insultation to reduce and remove environment-induced fluctuations. Broader applications of interferometric optical sensors in the future call for low-cost materials with both low thermal diffusivity and good soundproofing capability. In this paper, we explore the feasibility and effectiveness of natural soil as an insulation material for ultrahigh-resolution fiber-optic interferometry. An insulation chamber surrounded by soil is constructed, and its impact on the noise reduction of a Mach-Zehnder Fabry-Perot hybrid fiber interferometer is evaluated. Our results indicate that soil can effectively reduce ambient noise across a broad frequency range. Moreover, compared to conventional insulation materials such as polyurethane foam, soil shows superior insulation performance at low frequencies and thereby affords better long-term stability. This work demonstrates the practicability of soil as a legitimate option of insulation material for precision optical experiments.

## 1. Introduction

Ultrahigh-resolution optical interferometry has played a critical role in the advancement of modern science and technology, making impactful contributions such as the proof of a constant speed of light [1], the observation of gravitational waves [2], and the realization of Very Large Array (VLA) aperture synthesis [3]. The emergence of optical fibers over the last 40 years has brought tremendous interest in fiber-optic interferometry (FOI) [4,5]. Since light propagates through material (glass) rather than vacuum in FOI, the coupling between ambient environment and the light paths is stronger in FOI than in free-space interferometry. As a result, FOI is often highly susceptible to external perturbations such as temperature fluctuations and acoustic noises, which tend to mask the signals under investigation. This limits the resolutions of FOI in an array of applications including strain sensing [6], quantum optics [7], and remote clock distribution [8,9]. To overcome this barrier, high-quality thermal and acoustic insulations are frequently required.

Over the years, various insulating methods and materials have been developed to mitigate the impact of environmental disturbances. Highly specialized isolation chambers, such as anechoic chamber [10], thermostat chamber [11], and polyethylene-plasticine-lead-based chamber [12], have been utilized, and sophisticated insulation schemes such as impedance mismatching have been employed [13] in precision-measurement experiments. Most of these materials and schemes, however, are either of considerable cost or are complex in nature.

Meanwhile, little attention has been paid to low-cost, natural insulation materials. One such example is soil. Soil has been known for centuries as an excellent thermal and acoustic isolation material in construction and civil engineering [14], but has been largely overlooked in the context of precision optical measurement. Studies have found that the acoustical property of light-density soil is not strongly affected by its moisture content, allowing soil to attain a good acoustic absorption coefficient over a wide frequency range [15]. In fact, the acoustic-insulation capability of soil is comparable with or even better than some of the common soundproof materials such as fiberglass [15]. Soil also possesses a special property ideal for thermal insulation: it has both high thermal resistance and high thermal capacitance. This gives soil an especially low thermal diffusivity, which is defined as the ratio of thermal conductivity over volumetric heat capacity. In essence, thermal diffusivity measures how rapidly (instead of how much) heat flows within a material. For soil, thermal diffusivity typically ranges from 10^−8^ to 10^−7^ m^2^/s, which is comparable to some of the best thermal-insulating materials, such as polyurethane foams [16]. More importantly, unlike most other materials, soil enjoys an unparalleled cost-effectiveness due to its abundance in the nature.

In this paper, we report a systematic study on the effectiveness of natural soil as an environmental insulation material for high-precision fiber-optic interferometry. A high-resolution Mach-Zehnder Fabry-Perot (MZ-FP) hybrid fiber interferometer is utilized as the testbed, and its fluctuations are evaluated under various insulation conditions. Comparisons are made between the conventional polyurethane foam and a soil-based insulator in terms of their noise suppression capabilities. Our results demonstrate the feasibility of soil as a high-performance, low-cost insulation material for precision interferometric measurements.

## 2. A MZ-FP Hybrid Fiber Interferometer

In order to precisely gauge the insulation capability of soil, it is necessary to employ a sensor that is highly susceptible to ambient fluctuations. To this end, we have chosen to use a MZ-FP hybrid fiber interferometer. Details about the characterization of this interferometer have been reported elsewhere [17]. Here, we only give a brief overview of its operation principle for the convenience of the readers.

**Figure 1:**
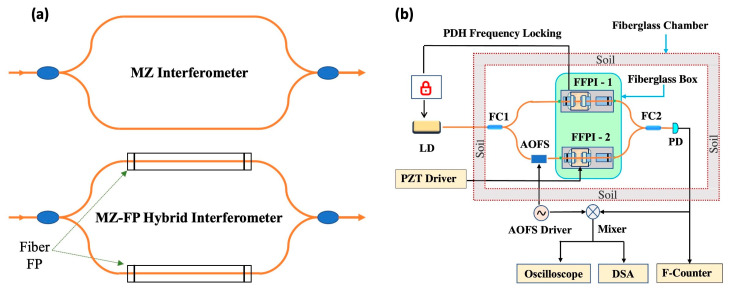
(**a**) The concept of the MZ-FP hybrid interferometer in comparison with the conventional MZ interferometer. (**b**) Schematics of the experimental setup. FC: fiber coupler, LD: laser diode.

Figure 1a illustrates the basic concept of the MZ-FP hybrid scheme. A normal Mach-Zehnder (MZ) interferometer consists of two arms, where light traverses once enroute to the output. In a MZ-FP hybrid fiber interferometer, two identical fiber Fabry-Perot (FP) interferometers (FFPIs) are inserted in the two arms of the MZ interferometer, forming a nestled configuration as shown in Figure 1a. The advantage of such a hybrid scheme is that an FFPI can effectively increase the optical path length without increasing the actual fiber length. As pointed out by Skolianos et al. [18], when operating on resonance, an FP cavity produces an effective path length (2/π)*F* times longer than the cavity length, where *F* is the finesse. This allows a MZ-FP hybrid interferometer to attain the sensing resolution of a long-arm interferometer while maintaining a compact size.

Of course, the benefits of this hybrid scheme do not come without tradeoffs. To ensure its proper operation, the interrogating laser must stay on resonance with both FFPIs for extended periods of time. This requires the use of Pound-Drever-Hall (PDH) frequency locking as well as a co-packaging scheme for the two FFPIs [17]. Figure 1b shows a schematic of the experimental setup. A notable feature of this system is the insertion of an acousto-optic frequency shifter (AOFS) in one of the MZ arms. The AOFS introduces a 50-MHz frequency shift between the two paths, leading to a 50-MHz beat note on the photodetector (PD). This beat note is further down-shifted on a mixer and analyzed by an oscilloscope (in the time domain) and a fast Fourier-transform (FFT) dynamic signal analyzer (DSA) (in the frequency domain). In addition, a frequency counter is utilized to measure the Allan deviation of the 50-MHz beat note for long-term stability characterization.

## 3. Noise Suppression by Soil-Based Insulation

Because of its long effective arm length (~600 m), the MZ-FP interferometer is extremely sensitive to tiny phase fluctuations induced by changes of the ambient conditions, making it suitable for evaluating the quality of insulations. As shown in Figure 1b, the two FFPIs are co-packaged in a fiberglass box. This box, along with the remaining parts of the MZ interferometer, is further sealed in a larger fiberglass chamber with inner dimensions of 42.5 cm × 31 cm × 26 cm and a wall thickness of 6.5 mm. The purpose of the chamber is to provide thermal and acoustic insulation to the entire interferometer. In our tests, different materials are attached to the inner walls of this insulation chamber, and the corresponding performances of the MZ-FP interferometer are assessed. All the tests are conducted under regular lab conditions, with an ambient temperature maintained at about 22 °C and a relative humidity level at about 40%.

**Figure 2:**
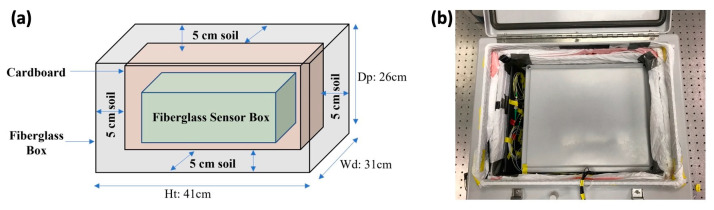
(**a**) Layout of the insulation chamber with the soil insulation layer. (**b**) A picture showing the inside of the insulation chamber.

As the first step, the effectiveness of soil-based insulation is quantified by comparing the output signals from the interferometer with and without the soil. The soil sample used in this research is a regular garden soil (containing mainly peat and compost) sold in department stores. It has a density of roughly 350 kg/m^3^ and a moisture level of about 25–30%. The thermal diffusivity of peaty soil is about 0.6 × 10^−7^ m^2^/s [19]. The acoustic absorption coefficient of soil typically ranges between 0.6 and 0.9 [15]. Figure 2a shows a layout of the insulation chamber with soil. Inside the chamber, a 5-cm-thick layer of the soil sample is filled up along each side of the box (including the top and bottom) to form a completely insulated environment at the center of the chamber. Airtight walls are built inside the soil layer to isolate the soil from the optical components so that no dirt or dust contaminates the optics. A picture of the setup is shown in Figure 2b, while the overall experimental system, including both the interferometer and the insulation system, is depicted in Figure 1b. Meanwhile, a comparison test has also been carried out with the soil layer removed from the fiberglass chamber. All other conditions remain the same between these two cases.

**Figure 3:**
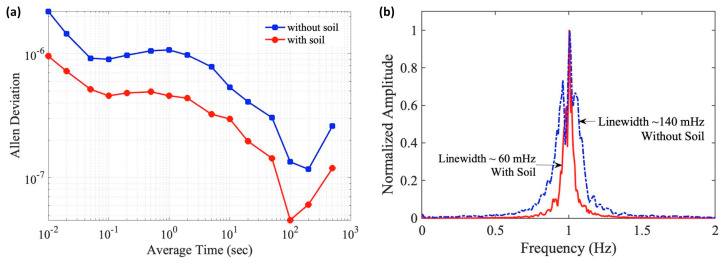
(**a**) The Allan deviations of the 50-MHz beat note from the MZ–FP interferometer with (circle) and without (square) the soil insulation. (**b**) The linewidths of the beat note (down–shifted to 1 Hz) with and without the soil insulation.

Figure 3a shows the measured Allan deviations of the 50-MHz beat note with (circle) and without the soil. Allan deviation characterizes the stability of a frequency source (the beat note in this case) under various time scales (i.e., the gate time of the frequency counter) [20]. Our result indicates that the addition of the soil layer leads to a near-uniform reduction of the Allan deviation across several orders of time scales. This suggests that soil is a broadband insulator with the ability to suppress both thermal (typically at low frequencies) and acoustic (normally at higher frequencies) fluctuations. Overall, with the 5-cm layer of soil, the average improvement of the Allan deviation across the entire measured time scales (0.01–500 s) is about 3.1 dB. In particular, the fractional frequency instability at a gate time of 1 s reduces from 1.07 × 10^−6^ without the soil to 4.58 × 10^−7^ with the soil, which represents a 3.6-dB improvement. Meanwhile, the spectrum of the beat note is measured by down-shifting the 50-MHz beat note to 1 Hz and inspecting it with the FFT DSA. The result is shown in Figure 3b. Again, the addition of the soil insulation results in the beat note linewidth reducing from 140 mHz to 60 mHz, a roughly 3.7-dB improvement, which agrees with the result from the Allan deviation measurement.

**Figure 4:**
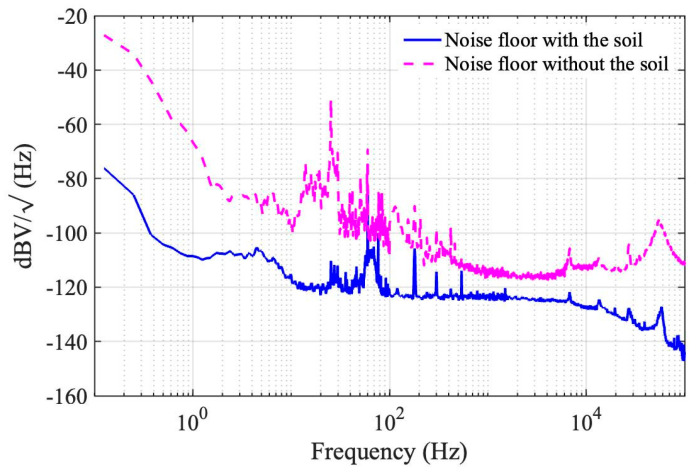
The noise spectra of the MZ–FP interferometer over a six—decade frequency span (0.1 Hz–100 kHz) with (solid) and without (dashed) the soil insulation.

Finally, the noise spectrum of the interferogram is directly characterized by mixing the beat note down to the baseband under the quadrature condition and measuring it with the DSA. Figure 4 shows the experimental traces with and without the soil over a 6-decade Fourier frequency range (10^−1^–10^5^ Hz). The result clearly indicates a broadband suppression of ambient noises by the soil. Especially, it has been shown that thermal-noise-limited fiber-optic sensing is achieved within a frequency range of 10^1^–10^5^ Hz [17], which highlights the crucial impact of the soil insulation to the operation of the MZ-FP hybrid interferometer.

## 4. Soil vs. Polyurethane Foam

**Figure 5:**
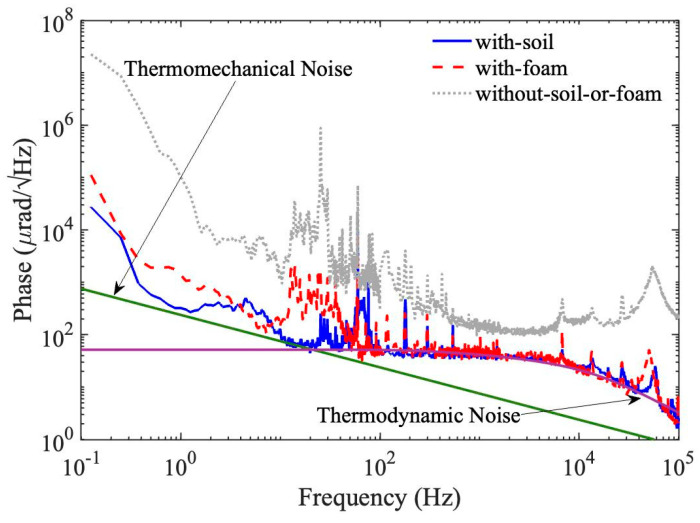
A comparison between the noise spectra of the MZ–FP interferometer with soil (solid) and with polyurethane foam (dashed) as the insulation material. The noise floor measured without either soil or foam is plotted as a reference. Additionally displayed are the theoretically predicted thermodynamic and thermomechanical noises due to spontaneous fluctuations inside the fibers [23].

To further evaluate the potential of soil as a cost-effective insulation material, we have performed a comparative study between soil and polyurethane foam. Polyurethane foam is a standard soundproof material, with a thermal diffusivity ranging between 4 and 6 × 10^−7^ m^2^/s [21] and a sound absorption coefficient of 0.9 (according to the manufacturer’s specifications). A second fiberglass chamber is constructed, where a 5-cm layer of polyurethane foam is adhered to the interior walls of all six sides. The MZ-FP interferometer is then transferred into this chamber, and the noise spectrum of the interferogram is measured to compare with the noise spectrum based on soil insulation. The result is shown in Figure 5. Additionally drawn in Figure 5 is the noise floor without either soil or foam, as well as the calculated thermal noise floors, including contributions from the thermodynamic noise and the thermomechanical noise [22]. These are theoretical predictions of the fundamental limits of interferometric fiber sensors due to intrinsic spontaneous fluctuations inside optical fibers [23]. The calculations of these noises are detailed elsewhere [17].

It is evident from Figure 5 that, between 100 Hz and 100 kHz, the MZ-FP interferometer is able to reach the thermodynamic noise limit with both the polyurethane foam and the soil. This suggests that, for a wide frequency range, soil can be as good an insulator as polyurethane foam. Below 100 Hz, the soil insulator generally performs better than the foam except for a narrow band around 60 Hz. Especially near the low-frequency end (i.e., <5 Hz), using the soil insulation clearly results in a noise floor much closer to the thermomechanical noise limit than using the polyurethane foam. This result is reasonable because (1) higher density (i.e., more mass) allows the soil layer to better isolate low-frequency acoustic waves than the foam, and (2) ambient temperature fluctuations mainly occur at the low-frequency end, and soil is a better thermal insulator than the polyurethane foam, as evident from its much lower thermal diffusivity. Overall, these comparisons demonstrate the superiority of soil over the commonly used polyurethane foam in terms of providing both thermal and acoustic insulations for precision optical measurement.

## 5. Conclusions

In conclusion, we have investigated the feasibility and effectiveness of natural soil as a thermal and acoustic insulation material for ultrahigh-resolution fiber-optic interferometry. The study is based on a MZ-FP hybrid fiber interferometer, whose noise characteristics are evaluated with and without the soil insulation. Comparisons are also made between soil and polyurethane foam in terms of their impacts on the noise spectra of the interferometer. Our results indicate that soil is highly effective in suppressing ambient noise across a broad frequency range. Moreover, compared to the commonly used polyurethane foams, soil performs especially well at low frequencies, which makes it an ideal choice for applications requiring long-term stability. This work demonstrates the feasibility of using natural soil to achieve cost-effective insulation in precision optical experiments.

## Data Availability

No new data were created or analyzed in this study. Data sharing is not applicable to this article.
